# On Sir Charles Bell's Researches in the Nervous System

**Published:** 1847-07-01

**Authors:** 


On Sir Charles Bell's Researches in the Nervous System. By *Alexander Shaw*, Surgeon to the Middlesex Hospital. 4to. pp. 40. Murray: London, 1847.

This Essay is appended to the posthumous edition of the well known "Anatomy and Philosophy of Expression, as connected with the Fine Arts." It contains a remarkably lucid exposition of Sir C. Bell's discoveries, written in a pleasing and very graceful style, and illustrated with many interesting facts drawn from the field of Comparative Anatomy. We must find room for one extract:—

"Man is distinguished above all other animals by his lower extremities having solidity and power sufficient to sustain his body without the aid of other members, and so as to be his sole organs of progression: hence his erect position. Again, as man's upper extremities are emancipated from the duty of assisting in locomotion, they are free to execute whatever rapid and varied movements may be called for, either for self-defence or for procuring nourishment. And, in correspondence with that freedom of action, a *Hand* is added, which, for the perfection of its endowments and mechanism, has been, in all ages, a constant theme of admiration.

"Now let us ask, what influence have the improvements thus shown in the construction of the organs of locomotion and of prehension, upon the structure of the Mouth? The chief use of the prehensile organs being to seize food for the supply of the mouth, it may be expected that, as they become more highly organized, the mouth will undergo a change in its form. What, then, is the effect of the improved organization of the prehensile organ, as seen in the hand of man, in allowing the mouth to be adapted for a vocal organ—an organ of Articulate Language?

"It has been stated that in all the vertebrate animals below man, the member analogous to the arm and hand, is an instrument of progression as well as of prehension. Whether we take the fin of the fish, the anterior extremity of the reptile, the wing of the bird, the paddle of the dolphin, or the fore leg of the horse or dog, the principal, if not the only use of the member is to assist in locomotion; only a few quadrupeds, like the squirrel, the feline animals, &c., besides using their paws for running, climbing, burrowing, &c., employ them to carry food to the mouth. Now the consequence of using the organ intended to convey the food to the mouth as one for progression, will be, that the office of appropriating the food will be thrown upon the mouth itself. Accordingly, in all animals below Man, the mouth is a prehensile, as well as a manducatory organ. If the animal be graminivorous, it must crop the herbage with its teeth before chewing and triturating it; if it be carnivorous, it must be provided with large, sharp fangs or tusks, to fight, seize, and tear its prey, before it can reduce its food to a fit state for swallowing. In short, the mouth, with its delicate sense of touch, its hairs or whiskers projecting from it as feelers, and its jaws armed with large teeth, is to be looked upon, in conjunction with the long, flexible neck commonly belonging to brutes, as combining the functions of the human arm and hand, with that of an organ of mastication.

"But it is obvious that a mouth of the large capacity and irregular shape of an animal like the horse, ox, dog, or lion, could never be adapted to produce articulate sounds. In a cavity such as the mouth of the horse, we can understand how neighing may be produced; but we cannot suppose that, by any adjustment of the tongue or lips, the air, even if it were properly vocalized in the larynx, could be confined, and then be let suddenly free to give rise to explosive sounds; or be impinged against the palate, to cause guttural sounds; or be directed into the back of the nostrils, to produce nasal sounds. In short, none of those numerous, finely-varied changes in the shape of the interior of the mouth, produced by the combined action of the tongue, palate, cheeks, and lips, which give rise to the infinite modifications of sound in speech, could take place in such gross structures. For the vibrating air expelled from the larynx to be divided and modulated so as to produce words with proper tone and accent, it is necessary that the cavity of the mouth should be small, its boundaries regular and uninterrupted, and the communication between it and the nostrils, free.

"Now that is the very character of the mouth in Man." P. 35.

